# Issues and advances in research methods on video games and cognitive abilities

**DOI:** 10.3389/fpsyg.2015.01451

**Published:** 2015-09-29

**Authors:** Bart Sobczyk, Paweł Dobrowolski, Maciek Skorko, Jakub Michalak, Aneta Brzezicka

**Affiliations:** ^1^GamesLab, Department of Psychophysiology of Cognitive Processes, Faculty of Psychology, SWPS University of Social Sciences and HumanitiesWarsaw, Poland; ^2^Institute of Psychology, Polish Academy of SciencesWarsaw, Poland

**Keywords:** video games, cognition, cognitive training, transfer of training, methodology

## Abstract

The impact of video game playing on cognitive abilities has been the focus of numerous studies over the last 10 years. Some cross-sectional comparisons indicate the cognitive advantages of video game players (VGPs) over non-players (NVGPs) and the benefits of video game trainings, while others fail to replicate these findings. Though there is an ongoing discussion over methodological practices and their impact on observable effects, some elementary issues, such as the representativeness of recruited VGP groups and lack of genre differentiation have not yet been widely addressed. In this article we present objective and declarative gameplay time data gathered from large samples in order to illustrate how playtime is distributed over VGP populations. The implications of this data are then discussed in the context of previous studies in the field. We also argue in favor of differentiating video games based on their genre when recruiting study samples, as this form of classification reflects the core mechanics that they utilize and therefore provides a measure of insight into what cognitive functions are likely to be engaged most. Additionally, we present the Covert Video Game Experience Questionnaire as an example of how this sort of classification can be applied during the recruitment process.

## Introduction

Video games are one of the most popular free-time activities, with 42% of Americans playing at least 3 h per week (Ipsos MediaCT, 2015). This growth has garnered the attention of many researchers, with numerous studies in the last decade showing the potential cognitive benefits of video gameplay. So far there have been over a 100 research reports comparing the performance of video game players (VGPs) and non-video game players (NVGPs) in cognitive tasks, as well as examples of cognitive enhancement after video game training (for a review see Mishra et al., 2012; for meta-analysis see Powers et al., 2013).

Despite progress in the field, some common methodological issues continue to persist (Boot et al., 2011; Schubert and Strobach, 2012; see Boot, 2015 for recent overview). This persistence stems from the fact that many researchers follow practices established by previous experiments without using a critical approach when considering the research methodology. VGPs are often arbitrarily defined as those players who spend a minimum of 5–7 h a week playing video games (Green and Bavelier, 2003, 2007). However, up to this point no studies have investigated the gameplay habits of VGPs in order to validate these criteria. Additionally, researchers focus primarily on “action video games” (AVGs), initially defined as “those that have fast motion, require vigilant monitoring of the visual periphery, and often require the simultaneous tracking of multiple targets” (Green and Bavelier, 2006). While setting such arbitrary criteria is not uncommon for early research within any field, recent findings indicate the need for their re-evaluation in order to explain the mechanisms of cognitive performance improvements as a consequence of gameplay experience. This is particularly problematic for training studies, as it is difficult to compare results from training regimes that use disparate treatments.

## What constitutes a video game player?

In many studies, VGPs are recruited based on “fairly simplistic, undifferentiated definitions of (video) game experience” (Boot, 2015). Unsworth et al. (2015) recently raised concerns regarding these inclusion criteria. They argued that most previous cross-sectional studies used extreme group designs that compared players with “significant video-game experience (typically 5+ h a week)” to NVGPs, and that this approach omits casual players. In their first study, a sample of VGPs (playing at least 5 h per week) outperformed NVGPs in symmetry span, fluid intelligence tests and attention-control, and showed a trend to outperform NVGPs on most other measures. However, their second study, which included the data of all VGPs with no minimum cut-off point for gameplay time, showed only four relatively weak correlations between video game experience and cognitive abilities. This result raises the important issue of how representative the commonly used recruitment criteria are of typical VGPs.

Addressing this issue, we analyzed a random sample of non-declarative data concerning gameplay times that was provided to us by the Valve Corporation. Their online platform Steam is a digital game distribution service for PC and Macintosh with over 125 million (Valve Corporation, 2015) active users worldwide and an estimated 75% of the global market for downloaded PC games (Edwards, 2013). Users launch their video games via Steam and their gameplay time is tracked individually for each game they own. We received a randomly selected and anonymously coded sample of 13,139 gameplay records collected over a period of 7 days from worldwide players. Entries with incorrectly registered data (i.e., duplicates) were excluded from analysis and averages were computed for player ID's that had multiple game entries. Accounts with 52 h or less of total gameplay time on their account (corresponding to 1 h of gameplay per week over a period of 12 months) were filtered out to remove new or unused accounts. Eight thousand, three hundred and thirty-five players were included in the final analysis.

**Figure 1:**
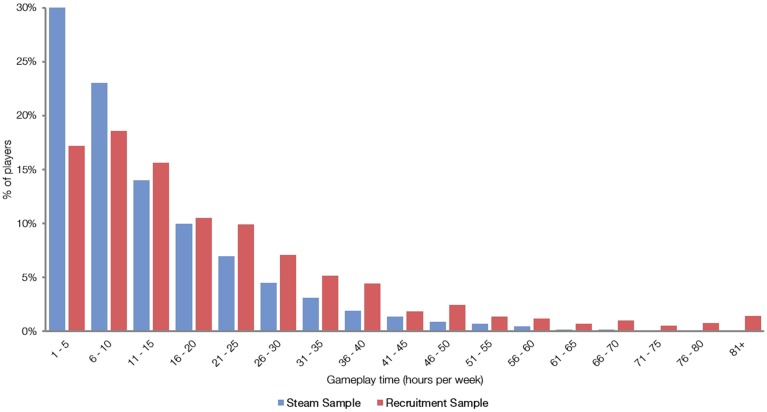
**Frequency distribution of Steam and recruitment samples' mean gameplay times**.

The data shows that this VGP sample played an average 13.45 h weekly with a *SD* of 12.85 (Figure 1). However, it is important to note that this data only represents the time spent playing video games through the Steam platform (PC market) and does not include playtime on other video game platforms (such as Origin or browser-based), devices (such as consoles or smartphones), or non-digitally purchased games. It is also possible for more than one player to use a single account (therefore possibly causing an overestimation of playtime), though this practice is inconvenient since they cannot do so simultaneously.

**Figure 2:**
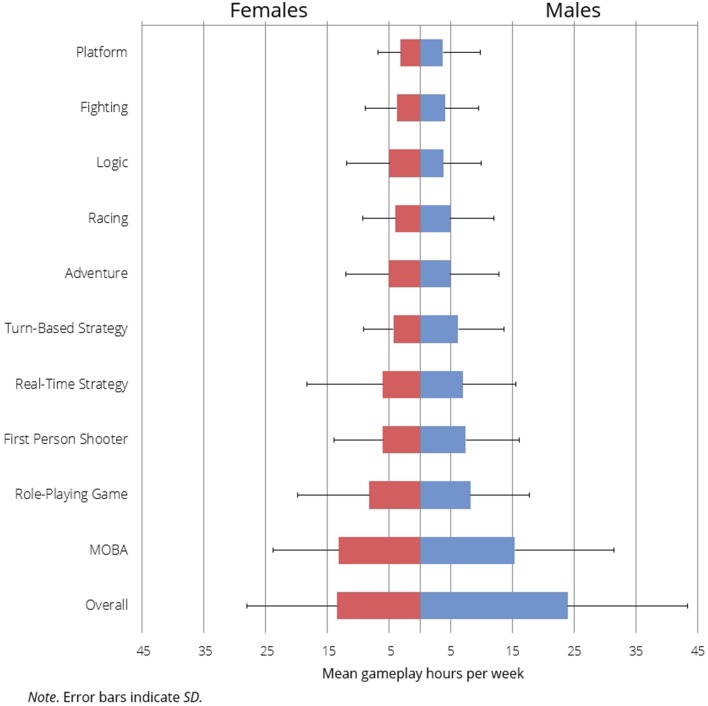
**Frequency distribution of recruitment sample's mean gameplay times for male and female participants per genres**.

In order to address these limitations, we analyzed the recruitment data of a general adult sample. Participants filled out a covert questionnaire containing multiple items related to free-time activities, including questions about the average number of hours spent playing video games per week in the preceding 6 months. For the purpose of this analysis we selected only participants who reported playing at least 1 h a week (*n*_excluded_ = 273) and removed those who reported playing more than 112 h per week (*n*_excluded_ = 4), assuming that this volume of play (16 h per day on average) is unlikely and unsustainable. The final sample included 1254 participants ranging in age from 18 to 64 (*M* = 24.48, *SD* = 6.86). Two hundred and sixteen of these participants reported playing 5 h or less, and 18 reported playing more than 80 h per week (see Figure 2 for a detailed distribution).

The data show that our sample played an average 21.06 h weekly (*Mdn* = 15 h) with a *SD* of 18.78. This suggests that video games are played more frequently on average than the generally adopted minimum recruitment criteria of 5–7 h weekly, though the playtime does vary substantially as suggested by Unsworth et al. (2015). It should be noted that while our recruitment was not directly targeted at VGPs, it was conducted in places that are more likely to be found by VGPs (internet announcement forums, university mailing lists, social media channels).

While we agree that the full range of gameplay experience should be taken into account when measuring cognitive enhancements, the approach taken by Unsworth et al. (2015) suffers from the very same flaws that they argue are present in video game research on supposedly extreme groups: the use of a sample that potentially represents an extreme of the overall playtime distribution. In this case it is a bias toward infrequent VGPs, the analysis of which unsurprisingly leads to small or no effects. Despite their full-range data, their participants' weekly gameplay frequencies were more representative of infrequent (6.5 h per week in their second study) than average VGPs (approximately 13.5 and 21.1 h per week for our Steam and recruitment samples, respectively). This was likely a strong contributing factor to their null findings.

Consequently, we cannot agree that the arguments and data presented by Unsworth et al. (2015) constitute evidence that video games may not lead to enhanced cognition. On the contrary, it appears that such enhancements are visible even when using infrequent VGPs in player/non-player comparisons (as shown by the authors themselves in their first study), and that researchers underestimate how much time VGPs spend playing games on average. The extreme groups approach usually utilizes samples selected from distant segments of the standard distribution, often basing on quartile split, where the upper and lower 25% of the distribution is selected for group comparisons (Preacher et al., 2005). While we agree that using extreme groups has its limitations, we do contend the notion that comparing samples of players with 5–7 h of gameplay time to non-players is representative of that approach.

We also conducted some additional analyses to describe the sample in more detail. Firstly, due to the previously mentioned limitation of the Steam sample, we verified whether our recruitment estimates of overall gameplay time across platforms were higher than those observed in the Steam sample. In order to quantify the evidence for our assumption, we compared the probabilities via the Bayes factor with JASP^1^ software (Love et al., 2015) for one-sided Bayesian independent samples hypothesis testing as recommended by Rouder et al. (2009), with a default Cauchy prior width of *r* = 0.707. The Bayes factor is *BF*_10_ = 4.16 × 10^69^; 95% CI: [−0.610, −0.490], decisively (Jeffreys, 1961) indicating higher overall gameplay time of our random sample (Figure 1).

There was also evidence for a number of additional interesting effects. First, women and men were found to decisively vary in their gameplay time (*BF*_10_ = 9.23 × 10^15^; 95% CI: [−0.696, −0.441]), with men playing on average 23.91 h per week (*SD* = 19.35) and women 13.42 h per week (*SD* = 14.66). Second, we analyzed players of each genre. Gender differences in gameplay times were not dependent on genre (for reports of all BF_01_ and CI please refer to the Supplementary Materials). However, the results for Turn-Based Strategies (*BF*_10_ = 1.94, 95% CI: [−0.453, 0.045]) indicated anecdotal evidence for differences between groups (Figure 2). Research of video gameplay habits indicates distinct genre preferences between males and females (Homer et al., 2012); the distinct preferences of our sample are shown in Supplementary Materials (Figure 1). Third, we found that gameplay time positively correlates with the number of used gaming devices (*BF*_+0_ = 2601; 95% CI: [0.074, 0.183]). Finally, both men and women in our sample were of similar age (*M*_male_ = 24.31, *SD* = 6.48; *M*_female_ = 24.88, *SD* = 8.08), and we found that overall gameplay time does not decrease with age (*BF*_0−_ = 18.75; 95% CI: [−0.073, 0.001]).

Subsequently, we would also like to point out the importance of gender in cognitive studies on video games. Because males and females differ in performance at various cognitive tasks (Halpern, 2013), it is also an important factor to be considered. Up to this time, many training studies still fail to use gender balanced samples (e.g., Glass et al., 2013), or fail to report any information about gender at all (e.g., Montani et al., 2014). This is especially troubling when VGP groups are comprised of primarily males while non-VGP groups are primarily female. For example, women have lower performance than men in mental rotation tasks (Kimura, 1999) and prefer non-mental rotation video games (Lucas and Sherry, 2004). However, gender differences can be reduced after training with a video game (Feng et al., 2007).

## Video games are not homogenous

Another important point is the way that VGPs are categorized in research. While some researchers perform their analyses on separate categories of games, there are a few problems with their approach. Researchers in the field do not claim that all video games have the potential to improve cognition, but generally those containing the elements described by Green and Bavelier (2006). There is little to no evidence of cognitive enhancement from many types of games, including those categorized as “Role Playing Games,” “Music” (Unsworth et al., 2015), or “Sports” (Achtman et al., 2008). The problem is that these categories are not based strictly on genres, and instead are often a mix of several types of games, e.g., first and third person shooters (Colzato et al., 2013; West et al., 2013; Wilms et al., 2013), shooters and role-playing games (Sungur and Boduroglu, 2012), sports and real time strategy (Gobet et al., 2014), first-person shooter, open world action-adventure, puzzle platformers, sports and racing games as a homogenous category (Buelow et al., 2015), a non-specific “action” category (Cain et al., 2012; McDermott et al., 2014), or no categories at all (Karle et al., 2010; Vallett et al., 2013; Kühn et al., 2014).

This leads to difficulty in comparing and interpreting results across studies, as cognitive changes from video gameplay might derive from the core game mechanics, such as types of stimuli, perspective, or pace. The recently proposed common demands hypothesis identifies this interaction as a possible mechanism accounting for video game related enhancements (Oei and Patterson, 2014), similarly to traditional cognitive trainings (Salminen et al., 2012) and laboratory tasks. A few researchers (Colzato et al., 2010; Boot et al., 2013; Ferguson, 2014) have previously speculated as to whether or not the results obtained from players are specific to the types of games they play, and some evidence for this explanation is beginning to surface (Dobrowolski et al., 2015; Oei and Patterson, 2015).

While we agree with Green and Bavelier (2015) that modern video games often include elements traditionally attributed to other genres, we disagree with their suggestion to depart from categorizing them. It is true that a genre-based classification is only generally descriptive due to the emergence of cross-genre games, but this is still much more descriptive of what the players experience than a very broad “action” category. In the case of cross-section comparisons, using VGPs that primarily play similar games is also a way to increase the homogeneity of samples. In the case of training studies, it is up to the experimenters to describe the training game as precisely as possible in terms of how its content and mechanics may affect their subjects.

As such, we strongly recommend categorizing video games according to their genre when planning and recruiting for future research. This is a standard practice in video game design (Apperley, 2006; Adams, 2009), which places video games into genres based on their game mechanics. For example, First Person Shooter (FPS) games (such as *Call of Duty, Counter-Strike, Battlefield*) are characterized by navigation in a three dimensional environment from the first person (egocentric) perspective, aiming and shooting coordination, and focus on the avatar and its surroundings, with physics often being similar to the real-world. Another popular genre, Real-Time Strategy (RTS, e.g., *StarCraft, Command, and Conquer*), requires players to view the environment from a top-down (allocentric) perspective and use strategic planning skills in order to manage multiple units within a visible portion of the environment, all while under time pressure. It is important to note that both of these genres qualify for the “action video game” category proposed by Green and Bavelier (2006) despite having dissimilar game mechanics. This practice increases the chance of combining very distinct video games into one category which, if the common demands theory has merit, leads to a limited potential for explaining the presence or lack of video game effects in research.

With well over a 100 research reports on potential benefits of video gameplay, these fundamental pitfalls make comparing the results between studies difficult. Powers et al. (2013) conducted such a comparison in a thorough meta-analytic review. Using random-effects models, they showed not only an advantage of VGPs over NVGPs, but also improvements in information processing in training studies. On other hand, training studies showed negligible effects on executive functioning, whereas cross-sectional studies revealed small to large effect sizes across domains. Notably, the authors also attempted to categorize the results by genre. However, we would like to point out that due to distinct recruitment practices and inconsistent genre classification across studies, drawing conclusions is susceptible to the omitted-variable bias where confounding variables may be present within distinct game core mechanics.

## The covert video game experience questionnaire

The importance of using covert recruitment for research was already underlined by Boot et al. (2011). While Schubert and Strobach's (2012) do point out that there is no evidence for a motivational effect on cognitive test performance in the video game literature, we would still recommend this practice when possible in order to minimize demand characteristics. For this reason, we developed a relatively short covert questionnaire that is designed to keep the objective of the study unknown to the participant.

The Covert Video Game Experience Questionnaire (see Supplementary Materials) consists of one block of questions about demographics and four blocks of questions about free-time activities (internet use, TV and cinema, video games, and physical activity). Each category begins with an initial filtering question about the frequency of a particular activity and is followed by more questions if the participant declares it to be more than once a week (with the exception of cinema being more than once a month). Participants are asked similar questions within each category, and the video game category is presented in the middle.

Information gathered from the questionnaire allow recruiters to determine mean weekly gameplay time in the preceding 6 months and gameplay time in individual genres (chosen by the participant), which allows for players from specific genres to be filtered and recruited. Additionally, the questionnaire collects data regarding years of video game experience, a topic that was recently the focus of research (Latham et al., 2015). The design of this questionnaire was aimed at addressing the methodological approaches of recruiting and categorizing VGPs in video game research. However, please note that the recruitment data presented earlier in this paper derives from a preliminary version of our questionnaire, thus not all of its items could be analyzed for the purpose of this manuscript.

## Final remarks

In our opinion, current research practices have benefitted greatly from the methodological discussions of recent years. However, the remaining issues are substantial. The way in which VGPs are defined and recruited likely does not represent the typical player that researchers aim to include in cross-sectional research. It seems that many VGPs spend more time on video games than initially thought, and that this tendency should be kept in mind when interpreting research results coming from player samples that were not well controlled. Additionally, in line with the common demands hypothesis, researchers should pay closer attention to the games that are most often played by their participants and classify them based on the similarity of their core mechanics. Following these recommendations can allow researchers to reduce the error coming from within-group variability in their samples and make finer predictions about the sources of cognitive enhancement resulting from video game training.

## Author contributions

BS developed the concept of the manuscript, contributed to the data acquisition, performed all data analyses and interpretation, and drafted the manuscript. PD, MS, JM, AB substantially contributed to the concept of the manuscript and provided critical revisions and changes to the manuscript. Additionally, PD contributed to drafting of the work and preparation of the Steam database and together with MS contributed to the data acquisition. All authors approved the final version of this manuscript for submission and agree to be accountable for all aspects of the work in ensuring that questions related to the accuracy or integrity of any part of the work are appropriately investigated and resolved.

### Conflict of interest statement

The authors declare that the research was conducted in the absence of any commercial or financial relationships that could be construed as a potential conflict of interest.

## Footnotes

^1^https://jasp-stats.org

## References

[B1] Achtman R. L. Green C. S. Bavelier D. (2008). Video games as a tool to train visual skills. Rest. Neurol. Neurosci. 26, 435–446. 10.1037/0893-164X.19.4.414 18997318 PMC2884279

[B2] Adams E. (2009). Fundamentals of Game Design, 2nd Edn. Berkley, CA: Pearson Education, Inc.

[B3] Apperley T. H. (2006). Genre and game studies. Simul. Gam. 37, 6–23. 10.1177/1046878105282278

[B4] Boot W. R. (2015). Video games as tools to achieve insight into cognitive processes. Front. Psychol. 6:3. 10.3389/fpsyg.2015.00003 PMC4300862 25653634

[B5] Boot W. R. Blakely D. P. Simons D. J. (2011). Do action video games improve perception and cognition? Front. Psychol. 2:226. 10.3389/fpsyg.2011.00226 21949513 PMC3171788

[B6] Boot W. R. Champion M. Blakely D. P. Wright T. Souders D. J. Charness N. (2013). Video games as a means to reduce age-related cognitive decline: attitudes, compliance, and effectiveness. Front. Psychol. 4:31. 10.3389/fpsyg.2013.00031 PMC3561600 23378841

[B7] Buelow M. T. Okdie B. M. Cooper A. B. (2015). The influence of video games on executive functions in college students. Comput. Hum. Behav. 45, 228–234. 10.1016/j.chb.2014.12.029

[B8] Cain M. S. Landau A. N. Shimamura A. P. (2012). Action video game experience reduces the cost of switching tasks. Attent. Percept. Psychophys. 74, 641–647. 10.3758/s13414-012-0284-1 22415446

[B9] Colzato L. S. van den Wildenberg W. P. Zmigrod S. Hommel B. (2013). Action video gaming and cognitive control: playing first person shooter games is associated with improvement in working memory but not action inhibition. Psychol. Res. 77, 234–239. 10.1007/s00426-012-0415-2 22270615

[B10] Colzato L. S. van Leeuwen P. J. van den Wildenberg W. P. Hommel B. (2010). DOOM'd to switch: superior cognitive flexibility in players of first person shooter games. Front. Psychol. 1:8. 10.3389/fpsyg.2010.00008 21833191 PMC3153740

[B11] Dobrowolski P. Hanusz K. Sobczyk B. Skorko M. Wiatrow A. (2015). Cognitive enhancement in video game players: the role of video game genre. Comput. Hum. Behav. 44, 59–63. 10.1016/j.chb.2014.11.051

[B12] Edwards C. (2013). Valve Lines Up Console Partners in Challenge to Microsoft, Sony. Bloomberg Business. Available online at: http://www.bloomberg.com/news/articles/2013-11-04/valve-lines-up-console-partners-in-challenge-to-microsoft-sony

[B13] Feng J. Spence I. Pratt J. (2007). Playing an action video game reduces gender differences in spatial cognition. Psychol. Sci. 18, 850–855. 10.1111/j.1467-9280.2007.01990.x 17894600

[B14] Ferguson C. J. (2014). Action game experimental evidence for effects on aggression and visuospatial cognition: similarities, differences, and one rather foolish question. Front. Psychol. 5:88. 10.3389/fpsyg.2014.00088 24570673 PMC3916832

[B15] Glass B. D. Maddox W. T. Love B. C. (2013). Real-time strategy game training: emergence of a cognitive flexibility trait. PLoS ONE 8:e70350. 10.1371/journal.pone.0070350 23950921 PMC3737212

[B16] Gobet F. Johnston S. J. Ferrufino G. Johnston M. Jones M. B. Molyneux A. (2014). ‘No Level Up!': no effects of video game specialization and expertise on cognitive performance. Front. Psychol. 5:1337. 10.3389/fpsyg.2014.01337 25506330 PMC4246654

[B17] Green C. Bavelier D. (2003). Action video game modifies visual selective attention. Nature 423, 534–537. 10.1038/nature01647 12774121

[B18] Green C. S. Bavelier D. (2006). Effect of action video games on the spatial distribution of visuospatial attention. J. Exp. Psychol. 32, 1465–1478. 10.1037/0096-1523.32.6.1465 17154785 PMC2896828

[B19] Green C. S. Bavelier D. (2007). Action video game experience alters the spatial resolution of vision. Psychol. Sci. 18, 88–94. 10.1111/j.1467-9280.2007.01853.x 17362383 PMC2896830

[B20] Green C. Bavelier D. (2015). Action video game training for cognitive enhancement. Curr. Opin. Behav. Sci. 4, 103–108. 10.1016/j.cobeha.2015.04.012

[B21] Halpern D. F. (2013). Sex Differences in Cognitive Abilities: 4th Edn. New York, NY: Psychology Press.

[B22] Homer B. D. Hayward E. O. Frye J. Plass J. L. (2012). Gender and player characteristics in video game play of preadolescents. Comput. Hum. Behav. 28, 1782–1789. 10.1016/j.chb.2012.04.018

[B23] Ipsos MediaCT (2015). The 2015 Essential Facts About the Computer and Video Game Industry. Entertainment Software Association. Available online at: http://www.theesa.com/wp-content/uploads/2015/04/ESA-Essential-Facts-2015.pdf

[B24] Jeffreys H. (1961). Theory of Probability. Oxford: Oxford University Press.

[B25] Karle J. W. Watter S. Shedden J. M. (2010). Task switching in video game players: benefits of selective attention but not resistance to proactive interference. Acta Psychol. 134, 70–78. 10.1016/j.actpsy.2009.12.007 20064634

[B26] Kimura D. (1999). Sex and Cognition. Cambridge, MA: MIT Press.

[B27] Kühn S. Lorenz R. Banaschewski T. Barker G. J. Büchel C. Conrod P. J.. (2014). Positive association of video game playing with left frontal cortical thickness in adolescents. PLoS ONE 9:e91506. 10.1371/journal.pone.0091506 24633348 PMC3954649

[B28] Latham A. J. Westermann C. Patston L. L. Tippett L. J. (2015). The care and testing of video-game players: using patterns of performance to provide insight into the effects of video-game experience and expertise. Front. Hum. Neurosci. Conference Abstract: XII International Conference on Cognitive Neuroscience (ICON-XII). 10.3389/conf.fnhum.2015.217.00280

[B29] Love J. Selker R. Marsman M. Jamil T. Verhagen A. J. Ly A. (2015). JASP (Version 0.7.1) [Computer Software].

[B30] Lucas K. Sherry J. L. (2004). Sex differences in video game play: a communication-based explanation. Commun. Res. 31, 499–523. 10.1177/0093650204267930

[B31] McDermott A. F. Bavelier D. Green C. S. (2014). Memory abilities in action video game players. Comput. Hum. Behav. 34, 69–78. 10.1016/j.chb.2014.01.018

[B32] Mishra J. Bavelier D. Gazzaley A. (2012). How to assess gaming-induced benefits on attention and working memory. Games Health J. 1, 192–198. 10.1089/g4h.2011.0033 24761314 PMC3833365

[B33] Montani V. De Filippo De Grazia M. Zorzi M. (2014). A new adaptive videogame for training attention and executive functions: design principles and initial validation. Front. Psychol. 5:409. 10.3389/fpsyg.2014.00409 24860529 PMC4026745

[B34] Oei A. C. Patterson M. D. (2014). Are videogame training gains specific or general? Front. Syst. Neurosci. 8:54. 10.3389/fnsys.2014.00054 24782722 PMC3986546

[B35] Oei A. C. Patterson M. D. (2015). Enhancing perceptual and attentional skills requires common demands between the action video games and transfer tasks. Front. Psychol. 6:113. 10.3389/fpsyg.2015.00113 25713551 PMC4322619

[B36] Powers K. L. Brooks P. J. Aldrich N. J. Palladino M. A. Alfieri L. (2013). Effects of video-game play on information processing: a meta analytic investigation. Psychon. Bull. Rev. 20, 1055–1079. 10.3758/s13423-013-0418-z 23519430

[B37] Preacher K. J. Rucker D. D. MacCallum R. C. Nicewander W. A. (2005). Use of the extreme groups approach: a critical reexamination and new recommendations. Psychol. Methods 10, 178–192. 10.1037/1082-989X.10.2.178 15998176

[B38] Rouder J. N. Speckman P. L. Sun D. Morey R. D. Iverson G. (2009). Bayesian t tests for accepting and rejecting the null hypothesis. Psychon. Bull. Rev. 16, 225–237. 10.3758/PBR.16.2.225 19293088

[B39] Salminen T. Strobach T. Schubert T. (2012). On the impacts of working memory training on executive functioning. Front. Hum. Neurosci. 6:166. 10.3389/fnhum.2012.00166 22685428 PMC3368385

[B40] Schubert T. Strobach T. (2012). Video game experience and optimized executive control skills -On false positives and false negatives: reply to Boot and Simons (2012). Acta Psychol. 141, 278–280. 10.1016/j.actpsy.2012.06.010

[B41] Sungur H. Boduroglu A. (2012). Action video game players form more detailed representation of objects. Acta Psychol. 139, 327–334. 10.1016/j.actpsy.2011.12.002 22266223

[B42] Unsworth N. Redick T. S. McMillan B. D. Hambrick D. Z. Kane M. J. Engle R. W. (2015). Is playing video games related to cognitive abilities? Psychol. Sci. 26, 759–774. 10.1177/0956797615570367 25896420

[B43] Vallett D. B. Lamb R. L. Annetta L. A. (2013). The gorilla in the room: the impacts of video-game play on visual attention. Comput. Hum. Behav. 29, 2183–2187. 10.1016/j.chb.2013.05.001

[B44] Valve Corporation (2015). Valve Announces Link, Source 2, SteamVR, And More At GDC. Available online at: http://store.steampowered.com/news/16000/.

[B45] West G. L. Al-Aidroos N. Pratt J. (2013). Action video game experience affects oculomotor performance. Acta Psychol. 142, 38–42. 10.1016/j.chb.2013.05.001 23220058

[B46] Wilms I. L. Petersen A. Vangkilde S. (2013). Intensive video gaming improves encoding speed to visual short-term memory in young male adults. Acta Psychol. 142, 108–118. 10.1016/j.actpsy.2012.11.003 23261420

