# Supplementary material for: Issues and advances in research methods on video games and cognitive abilities
Source: Front Psychol. 2015 Sep 29;6:1451. doi: 10.3389/fpsyg.2015.01451 (PMC4586355; doi:10.3389/fpsyg.2015.01451)
Supplement: Supplementary file 1 [file Table1.DOCX]

***Supplementary Material***

**Issues and Advances in Research Methods on Video Games and Cognitive Abilities**

**Bart Sobczyk^1,^*, Paweł Dobrowolski^2^, Maciek Skorko^2^, Jakub Michalak^1^, Aneta Brzezicka^1^**

^1^ Department of Psychophysiology of Cognitive Processes at Faculty of Psychology, SWPS University of Social Sciences and Humanities, Warsaw, Poland

^2^ Institute of Psychology, Polish Academy of Sciences, Warsaw, Poland

*** Correspondence:** Bart Sobczyk, GamesLab S105, University of Social Sciences and Humanities, Chodakowska 19/31, 03-815 Warsaw, Poland

science@sobczyk.info

1. **Gender Comparison of Gameplay Time per Genre**

*Table 1.* Bayesian Independent Samples T-Test for the null hypothesis that men and women do not differ in their gameplay times per individual genres.

| Genre | BF_01_ | CI 95% |
| --- | --- | --- |
| FPS | 2.840 | -.364, .051 |
| Platform | 5.185 | -.303, .086 |
| Fighting | 6.357 | -.302, .185 |
| Adventure | 9.115 | -.170, .209 |
| RTS | 5.708 | -.324, .272 |
| RPG | 5.155 | -.099, .315 |
| Racing | 3.521 | -.311, .048 |
| Logic | 1.126 | .017, .324 |
| MOBA | 3.459 | -.558, .306 |

*Note.* Above list does not include data for Turn Based Strategies that is reported in the manuscript.

*Figure 1.* Frequency distribution of VGPs sample playing at least 1 hour per week of particular genre.

*Note.* MOBA category may be underrepresented as it was included during data collection.

1. **Covert Video Game Experience Questionnaire**

This questionnaire is designed to collect information about video game experience in a covert structure as an element of the recruitment process. Each category of questions (Demographics, Internet, TV and Cinema, Video Game Expertise, Sport and Physical Activities) is displayed on a separate page. The demographics category may include additional questions when relevant. Answering the initial question on the frequency of a particular activity determines whether following questions within the category are displayed.

An online version of this questionnaire is available to view at: http://gex.net.pl/vrlab/run/CVGEQ.

Instruction:

We would like to learn more about your free-time activity habits by asking about a number of common ways of spending time. It may take you 5-15 minutes to answer all of the questions. Please make sure that all answers are true and represent your habits.

Important note:

If you are unsure about your answers (such as the precise time committed to each activity), please provide your best estimate.

*Table 2.* Covert Video Game Experience Questionnaire items.

| No. | Item |
| --- | --- |
| Demographics | |
|  | Gender |
|  | - 1. Male   2. Female |
|  | Date of birth |
| Internet | |
|  | How often do you use the internet?^a^ |
|  | - 1. Once a month or less   2. More than once a month   3. More than once a week   4. Once a day   5. More than once a day |
|  | In the past 6 months how many hours per week did you use internet on average? |
|  | For what purpose do you use the internet most often?^b^ |
|  | 1. Collecting information 2. Communication 3. Education 4. Entertainment 5. Shopping 6. Work 7. Other (please specify) |
|  | On which device do you use the internet most frequently? ^b^ |
|  | - 1. PC, laptop, netbook, Mac   2. Tablet (iPad, Android etc.)   3. Smartphone (iPhone, Blackberry, Android phone, etc.)   4. Console (PlayStation, Xbox, Wii etc.)   5. Ebook reader (Kindle, Nook etc.)   6. Other (please specify) |
|  | In the past 6 months, how many hours per week of internet use on average did you spend on following activities? |
|  | Hours distribution sliders based on values from item 4 and answers from item 5. ^c^ |
| Television and Cinema | |
|  | How often do you watch TV? ^a^ |
|  | 1. Once a month or less 2. More than once a month 3. More than once a week 4. Once a day 5. More than once a day |
|  | How often do you go to the cinema? ^a^ |
|  | 1. Once a month or less 2. More than once a month 3. More than once a week 4. Once a day 5. More than once a day |
|  | In the past 6 months, how many hours per week on average did you watch TV? |
|  | What kind of programs do you watch most often? ^b^ |
|  | 1. Concerts, events 2. Documentaries 3. Movies 4. Music 5. News 6. Quiz shows, reality TV 7. Sport 8. TV series 9. Other (please specify) |
|  | Please list the titles of 3 TV programs that you watched most often in the past 6 months. |
|  | Which genres of movies or TV shows do you watch most often? ^b^ |
|  | 1. Action 2. Adventure 3. Comedy 4. Crime 5. Fantasy 6. Historical 7. Historical fiction 8. Horror 9. Mystery 10. Philosophical 11. Political 12. Romance 13. Science fiction 14. Thriller 15. Other (please specify) |
|  | In the past 6 months, how many hours per week on average did you spend watching the following types of programs? |
|  | Hours distribution sliders based on values from item 10 and answers from item 11. ^c^ |
|  | In the past 6 months, how many movies did you see in the cinema? |
| Video Games | |
|  | How often do you play video games? ^a^ |
|  | 1. Once a month or less 2. More than once a month 3. More than once a week 4. Once a day 5. More than once a day |
|  | On which device do you play video games most often? ^b^ |
|  | 1. PC, Mac, laptop 2. Console (PlayStation, Xbox, Wii etc.) 3. Tablet or smartphone (iPhone, Blackberry, Android phone etc.) 4. Other (please specify) |
|  | In the past 6 months, how many hours per week on average did you spend on playing video games? |
|  | Please rate your overall experience level with video games: |
|  | 1 – 7 Likert scale: 1 – Not experienced at all, 7 – Very experienced |
|  | For how many years have you played video games?^d^ |
|  | In the past 6 months, which video game genres did you play most often? ^b^ |
|  | 1. Adventure (*Zelda, Heavy Rain, The Wolf Among Us, Broken Sword,* etc.) 2. Fighting (*Street Fighter, Tekken, Virtual Fighter, Dead or Alive,* etc.) 3. First Person Shooters (*Call of Duty, Counter-Strike, Battlefield, Medal of Honor,* etc.) 4. Logic/Puzzle/Cards/Quiz (*Angry Birds, Lemmings, Solitaire, Hearthstone,* etc.) 5. Multiplayer Online Battle Arena (*League of Legends, Dota 2, Warhammer Online, World of Tanks,* etc.) 6. Open World Action-Adventure (*Grand Theft Auto, Red Dead Redemption, Saints Row, Sleeping Dogs,* etc.) 7. Platform (*Mario, Donkey Kong, Rayman, Trine,* etc.) 8. Racing (*Need for Speed, Gran Turismo, Forza, Mario Kart,* etc.) 9. Real-Time Strategies (*StarCraft, Command & Conquer, Age of Empires, Earth,* etc.) 10. Role-Playing Games (*Final Fantasy, Dragon Age, World of Warcraft, Diablo,* etc.) 11. Simulation (*SimCity, The Sims, Tropico, Transport Tycoon,* etc.) 12. Sport (*EA Sports FIFA, Pro Evolution Soccer, NBA Live, Mario & Sonic at the Olympic Games,* etc.) 13. Third Person Shooters (*Metal Gear Solid, Dead Space, Gears of War, Resident Evil,* etc.) 14. Turn-Based Strategies (*Civilization, Heroes of Might and Magic, Total War Series, Panzer General,* etc.) 15. Vehicle simulation (*Microsoft Flight Simulator, Euro Truck Simulator, Train Simulator, Silent Hunter,* etc.) 16. Other (please specify) |
|  | Please list 3 video game titles that you played most often in the past 6 months. |
|  | Please rate how good you are in following genres: |
|  | 1 – 7 Likert scale: 1 – Not good at all, 7 – Very good; only genres selected in item 21 appear |
|  | In the past 6 months, how many hours per week on average did you spend playing each genre? |
|  | Hours distribution sliders based on values from item 18 and answers from item 21. ^c^ |
|  | In the past 6 months, how many hours per week on average did you spend playing each device? |
|  | Hours distribution sliders based on values from item 18 and answers from item 17. ^c^ |
| Sport and Physical Activities | |
|  | How often do you do engage in sports or similar physical activities? ^a^ |
|  | 1. Once a month or less 2. More than once a month 3. More than once a week 4. Once a day 5. More than once a day |
|  | In the past 6 months, how many hours per week on average did you spend on sports or similar physical activities? |
|  | In the past 6 months, which sports or similar activities do you spend the most time on? |
|  | In the past 6 months, how many hours per week on average did you spend on each sport or physical activity? |
|  | Hours distribution sliders based on values from item 27 and answers from item 28. ^c^ |
|  | Please rate how good you are at the following activities: |
| End of questionnaire | |
|  | If there is anything else you would like to tell us, please let us know below. |

*Note.* No answers indicate an open-ended question. ^a^ Initial filtering question presented individually on page. Following questions in category will appear only if participant frequently participates in the activity (see Fig. 1). ^b^ Multiple answers may be selected. ^c^ Participant sees a total number of hours declared and distributes them between the answers with intervals of 30 minutes. ^d^ Answer should not exceed the age of participant.

*
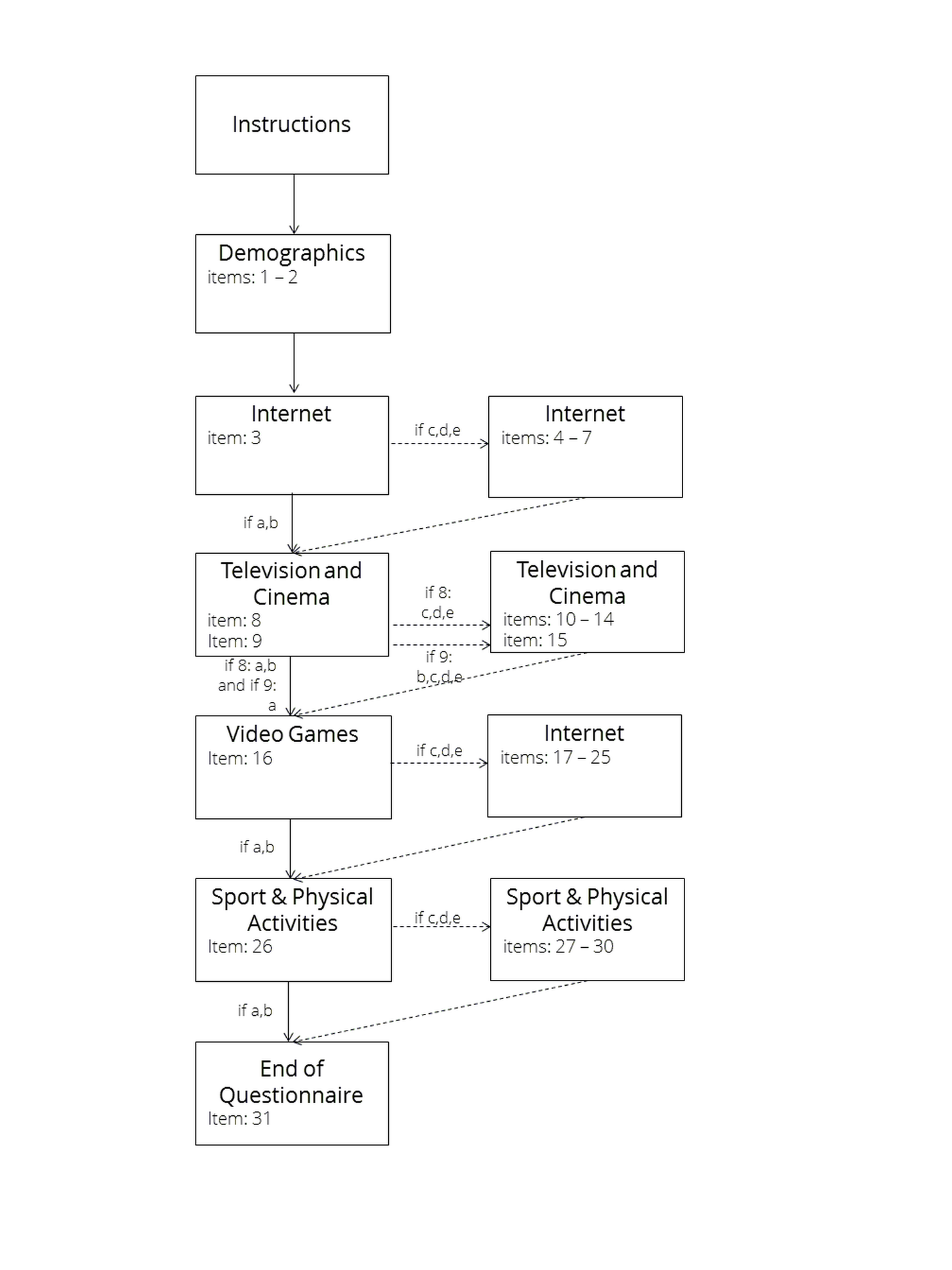
*

*Figure 2.* Diagram of Covert Video Game Expertise Questionnaire pathway.
